# Dataset of nine agronomic traits in bread wheat phenotyped under irrigated and rain-fed environments

**DOI:** 10.1016/j.dib.2022.107933

**Published:** 2022-02-10

**Authors:** Vijay Gahlaut, Vandana Jaiswal, Bhudeva S. Tyagi, Gyanendra Singh, Sindhu Sareen, Harindra S. Balyan, Pushpendra Kumar Gupta

**Affiliations:** a Department of Genetics and Plant Breeding, Ch. Charan Singh University, Meerut, India; b ICAR-Indian Institute of Wheat and Barley Research, Karnal, India

**Keywords:** Drought, Doubled haploid, Breeding, Wheat

## Abstract

Higher yield and broad adaptation to drought-prone environments are key targets of wheat breeding programs. This can be achieved through a complete knowledge of the genetic architecture of yield and its related traits. This brief article provides analysed mean data used in the research article entitled “QTL mapping for nine drought-responsive agronomic traits in bread wheat under irrigated and rain-fed environments” (Gahlaut et al., 2017). Phenotypic data were recorded on nine important agronomic traits on a doubled haploid (DH) mapping population derived from the cross Kukri/Excalibur. For recording this data, the mapping population was grown during three crop seasons (2010–11 to 2012–13) at four separate locations in India, both under irrigated and rain-fed environments. This dataset is valuable for wheat breeders to better understand the genetic basis of drought tolerance in wheat.

## Specifications Table

**Table:** 

Subject	Agricultural Sciences
Specific subject area	Agronomy and Crop Science
Type of data	Tables
How the data were acquired	Data were acquired from observation, measurements, and sampling in the field and after harvesting
Data format	Analysed mean data
Description of data collection	A mapping population involving 192 doubled haploid (DH) lines was grown at four locations under irrigated (IR) and rain-fed (RF) conditions over three crop seasons (2010–11 to 2012–13). The data were recorded on following nine agronomic traits (i) germination percentage (GP), (ii) days to anthesis (DTA), (iii) days to maturity (DTM), (iv) grain filling duration (GFD), (v) plant height (PH), (vi) grain weight/ear (GWPE), (vii) productive tillers/m^2^ (PTPM), (viii) 1000-grain weight (TGW) and (ix) grain yield per plot (GYPP)
Data source location	Provided in Table 1
Data accessibility	The dataset is provided as Microsoft Excel (.xlsx). The dataset is available on this article and can be found in Mendeley repository data: https://data.mendeley.com/datasets/g2fyhm53p9/1
Related research article	[1] V. Gahlaut, V. Jaiswal, B.S. Tyagi, G. Singh, S. Sareen, H.S. Balyan, P.K. Gupta, QTL mapping for nine drought-responsive agronomic traits in bread wheat under irrigated and rain-fed environments, PLoS ONE. 12(2017) e0182857. https://doi.org/10.1371/journal.pone.0182857

## Value of the Data

• The phenotypic data presented here provides a reference to the agronomical performance of wheat grown under different water regimes.
• Agronomy and crop science researchers can benefit from these datasets.
• This dataset includes a wide range of agronomical traits that were phenotype at four locations. It can be used for further genetic studies and breeding programs of wheat, especially for drought tolerance.

## 1 Data Description

This article contains analysed mean data for nine agronomic traits recorded on a mapping population consisting of 192 DH lines. The experiment was carried out at four locations under irrigated (IR) and rain-fed (RF) conditions over three crop seasons during 2010/11 to 2012/13. The details of experimental locations and other related information are provided in Table 1. The analysed mean data of following nine agronomic traits: (i) germination percentage (GP), (ii) days to anthesis (DTA), (iii) days to maturity (DTM), (iv) grain filling duration (GFD), (v) plant height (PH), (vi) grain weight/ear (GWPE), (vii) productive tillers/m^2^ (PTPM), (viii) 1000-grain weight (TGW) and (ix) grain yield per plot (GYPP) is available at Mendeley Data (https://data.mendeley.com/datasets/g2fyhm53p9/1 [2]).

**Table 1:** Field experiments and locations details.

Experiment location	Latitude	Longitude	Altitude (m)
Chaudhary Charan Singh Haryana Agricultural University, Hisar, Haryana, India	29.15°N	75.70°E	215
Chandra Shekhar Azad University of Agriculture and Technology, Kanpur, Uttar Pradesh, India	26.27°N	80.14°E	126
Indian Institute of Wheat and Barley Research, Karnal, Haryana, India	29.68°N	76.98°E	227
Agharkar Research Institute, Pune, Maharashtra, India	18.31°N	73.52°E	560

## 2 Experimental Design, Materials and Methods

### 2.1 Plant material

A double haploid (DH) mapping population was developed by crossing drought tolerant and sensitive genotypes. Excalibur and Kukri were used as male and female parent respectively to develop mapping population. Excalibur is a drought tolerant cultivar and was released in 1999 and Kukri is a hard-white wheat and drought sensitive cultivar and, was released in 1991 [3].

This DH population contained a set of 192 DH lines.

### 2.2 Experimental design

For phenotypic evaluation, DH lines were grown at four locations in India, i.e. Hisar, Kanpur, Karnal and Pune for three consecutive crop seasons, i.e., 2010–11, 2011–12 and 2012–13 (For more details, see Table 1). At each location, experiment was conducted under IR and RF conditions. Augmented experimental design was used to raise the 192 DH lines along with three check genotypes (NI5439, PBW175, and WH147). The experimental design includes 12 blocks; each block contained 19 entries, including 16 DH lines and three check genotypes. Each line in a block consisted of a plot of three rows, each row of 1.5 m length, with row-to-row distance of 25 cm. The seed rate was 10 g seed/m^2^ for each genotype. Standard agronomic practices were followed for conducting the experiments. In IR environments, four flood irrigations (each with 45 mm) in addition to rainfall were given. The first irrigation was given at 21 days after sowing (DAS), subsequently, second, third and fourth irrigations were given at 40, 60 and 80 DAS, respectively (Table 2). In the RF environments, single irrigation was given at 21 DAS to allow the crop to establish and to avoid complete crop failure. Harvesting was done in late March or early April in each crop-season, to prevent the experience of heat stress in late April. The details of climatic data (rainfall, total daylight, day length) during three crop seasons at four locations in India are provided in Table 2.

**Table 2:** Growing season climatic data for the field experiments.

			Day length (hours)	
Experiment	Rainfall# (mm)	Total daylight hours	minimum	maximum
Hisar (2010–11)-IR	254	1650	10.3	12.8
Hisar (2010–11)-RF	74	1650	10:03	12.8
Hisar (2011–12)-IR	352	1611	10:03	12.7
Hisar (2011–12)-RF	172	1611	10:03	12.7
Kanpur (2010–11)-IR	282	1499	10:05	12.5
Kanpur (2010–11)-RF	102	1499	10.5	12.5
Kanpur (2011–12)-IR	282	1561	10.5	12.5
Kanpur (2011–12)-RF	102	1561	10.5	12.5
Kanpur (2012–13)-IR	367	1592	10.5	12.5
Kanpur (2012–13)-RF	187	1592	10.5	12.5
Karnal (2010–11)-IR	251	1530	10.2	12.5
Karnal (2010–11)-RF	71	1530	10.2	12.5
Karnal (2011–12)-IR	264	1477	10.2	12:06
Karnal (2011–12)-RF	84	1477	10.2	12.6
Karnal (2012–13)-IR	440	1550	10.2	12.8
Karnal (2012–13)-RF	260	1550	10.2	12.8
Pune (2010–11)-IR	314	1476	11	11.7
Pune (2010–11)-RF	134	1476	11	11.7
Pune (2011–12)-IR	282	1535	11	11.8
Pune (2011–12)-RF	102	1535	11	11.8
Pune (2012–13)-IR	250	1547	11	11.5
Pune (2012–13)-RF	70	1547	11	11.5

# A combined estimate of rainfall and the water volume applied using flood irrigation. IR, irrigated; RF, rain-fed.

### 2.3 Data collection

The DH lines were phenotyped and data were recorded for the following nine traits –

(i) GP: Germination was considered when radical with >2 mm length was emerged from the soil. Number of germinated seed were counted and GP was calculated with the formula GP = (number of germinated seed/total seed sown) x 100. The germination percent in each plot was recorded daily up to 10 days after sowing.
(ii) DTA: For DTA, date on which anthers were extruded in >75% spikes of individual plot was recorded, now days were calculated from date of sowing to extrusion of anthers and considered as DTA.
(iii) DTM: Similar to DTA, for DTM, date on which >75% spikes of plot became pale yellow (sign of physiological maturity) was recorded; and DTM was calculated as days from sowing to maturity date.
(iv) GFD: GFD was calculated from DTA to DTM data. Number of days between DTA to DTM was considered as GFD.
(v) PH: At the time of anthesis, height of the five representative random tillers in plots of each genotype were measured in cm from the surface of the soil to the tip of the spike excluding awns and their average was recorded as plant height.
(vi) GWPE: After harvesting, seed of five representative random spikes from each plot were harvested individually and weight in g. Mean weight of five spikes were calculated as GWPE.
(vii) PTPM: One meter square area randomly selected in each plot and number of ear bearing tillers were counted. The process was repeated three times with random one meter square area and mean of three replicates was considered as PTPM.
(viii) TGW: Random 1000-grains of each genotype were counted and weighed in g and recorded as 1000-grain weight.
(ix) GYPP: Plant material of whole individual experimental plots of each genotype was harvested and threshed to obtain the grains that were weighed in g.

### 2.4 Statistical data analysis

The raw data of all nine traits at in all the environmental conditions (available at https://figshare.com/articles/dataset/QTL_mapping_for_nine_drought-responsive_agronomic_traits_in_bread_wheat_under_irrigated_and_rain-fed_environments/5295943 [4]) were checked for normality and homogeneity. Augmented experimental design is a non-replicated design and checks are used to nullify the experimental error. During present study, the adjusted mean of 192 DH lines for nine traits in all environments was estimated using three check varieties, and for this purpose Statistical Package for Augmented Designs (SPAD) package [5] was used.

## Ethics Statements

The paper is not currently being considered for publication elsewhere.

## CRediT authorship contribution statement

**Vijay Gahlaut:** Conceptualization, Data curation, Writing – original draft. **Vandana Jaiswal:** Data curation, Writing – original draft. **Bhudeva S. Tyagi:** Resources, Data curation. **Gyanendra Singh:** Resources, Data curation. **Sindhu Sareen:** Resources, Data curation. **Harindra S. Balyan:** Supervision, Conceptualization, Writing – review & editing. **Pushpendra Kumar Gupta:** Supervision, Conceptualization, Writing – review & editing.

## Declaration of Competing Interest

The authors declare that they have no known competing financial interests or personal relationships that could have appeared to influence the work reported in this paper.
